# Dezocine and Addiction: Friend or Foe?

**DOI:** 10.3390/ph18030386

**Published:** 2025-03-08

**Authors:** Wayne Childers, Khaled Elokely, Magid Abou-Gharbia

**Affiliations:** 1 Department of Pharmaceutical Sciences, School of Pharmacy, Temple University, Philadelphia, PA 19140, USA; wayne.childers@temple.edu; 2 Department of Pharmaceutical Sciences, School of Pharmacy, College of Health Sciences, University of Wyoming, Laramie, WY 82071, USA; kelokely@uwyo.edu

**Keywords:** dezocine, addiction management, partial G-protein biased MOR agonist, NRI reuptake inhibition

## Abstract

The neurological effects of opium were first described over 8000 years ago. Morphine was isolated in 1803 and by the mid-1800s had become both a pain-relieving blessing and an addictive curse. As part of the crusade to identify safer and more reliable alternatives to morphine, dezocine (Dalgan^®^) was marketed in the US in 1986. Its use was discontinued in the US in 2011 without revealing the reasons, but it remains one of the most widely used analgesic agents in China today. Dezocine’s unique pharmacology makes it an effective analgesic with limited opioid-associated side effects and little or no reported potential for dependence and addiction. In addition, dezocine’s blocking effect on serotonin and norepinephrine transporters recommends its further exploration as a potential treatment for various chronic and neuropathic pain conditions. Most recently, data suggest that dezocine might represent a viable treatment for addiction management. This report focuses on the data supporting dezocine’s non-addictive profile and its potential use to treat opioid addiction and withdrawal, as well as recent efforts to generate formulations of dezocine that support sub-chronic and chronic dosing.

## 1. Introduction

**Figure 1:**
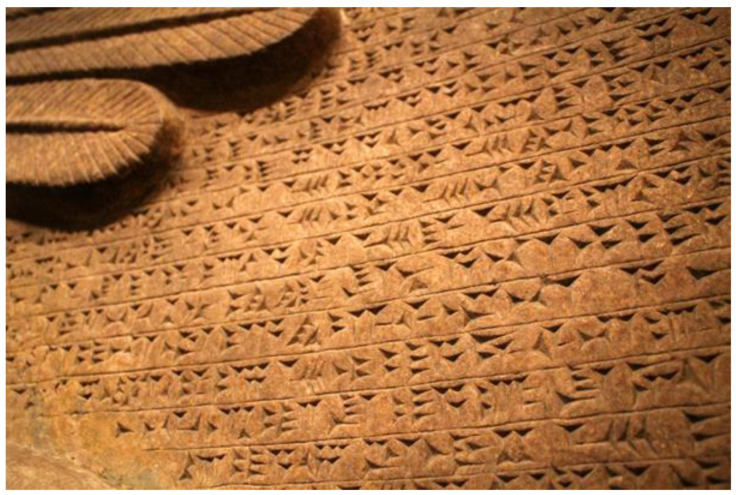
Written records describing opium date back over 5000 years to Sumarian clay tablets from Nippur, Iraq. Used under license from Shutterstock.

The first written records mentioning opium date back over 5000 years to Sumarian clay tablets [1,2] found at the site of the ancient city of Nippur in present-day Iraq (Figure 1). Its use was documented throughout the Bronze Age, the Middle Ages, and the Renaissance period and was mentioned in historic literature such as Homer’s The Odyssey, the writings of Hypocrates, and the Bible. The discovery of morphine as the active analgesic ingredient in opium is credited to the German pharmacist’s assistant Friedrich William Adam Serturner, who isolated it as a gummy substance from opium poppies in 1803 and named it after Morpheus, the Greek god of dreams [3]. Soon afterwards, morphine became a mainstay of pain treatment. The invention of the hypodermic needle in 1853 facilitated the use of morphine to treat neuralgia associated with surgical procedures, but the social use of opium was still prevalent. By the 1830s, the addictive liability of morphine was recognized, and the US government began a campaign to reduce the social use of morphine and opium and limit their uses to medicinal applications, culminating in the Opium Exclusion Act of 1909 and the Harrison Narcotics Tax Act of 1914. Drug discovery around morphine began with the synthesis of heroin in 1874 [4], but it became apparent that many of the semisynthetic analogs derived from morphine retained an addiction liability.

**Figure 2:**
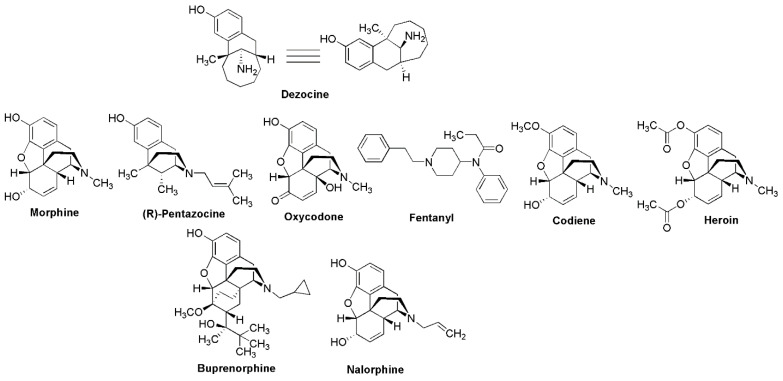
Structures of dezocine and the opioid agonists, partial agonists, and antagonists discussed in this paper.

Opioid use disorder (OUD) affects more than 16 million people worldwide [5]. Deaths from opioid overdose in the US alone rose to an astonishing 81,806 in 2022 [6]. That number has decreased somewhat in the past two years thanks to the use of mu opioid antagonists/partial agonists like buprenorphine, methadone, naloxone, and naltrexone (especially long-acting agents or extended release formulations), but it is a commonly held position within the medical community that not enough patients are receiving these drugs. Patients who use opioids chronically, either socially or for the treatment of pain, are at high risk for becoming addicted to those agents. Buprenorphine (Figure 2) has become the most widely used treatment for analgesic opioid addiction and withdrawal [7], but buprenorphine itself can cause addiction and withdrawal, and patients must be tapered off of it just like mu opioid full agonists. Some patients remain on buprenorphine chronically or indefinitely.

Dezocine (Dalgan^®^, Figure 2) is an aminotetralin derivative that shares structural similarity with the benzomorphan opioid R-pentazocine (Figure 2). It was discovered at the Wyeth division of American Home Products (WY-16,255) and first disclosed as an analgesic agent in 1973 [8] with nearly 3-fold greater analgesic potency compared to morphine. Dezocine was marketed in 1986 but was voluntarily withdrawn from the US market in 2011. No official reason for the withdrawal was given, although it is conceivable that decreasing sales and liability associated with the growing opioid crisis may have played a role in the decision [9]. However, dezocine remains one of the most frequently used opioid analgesic agents in China to this day. Until recently, its use was limited to postsurgical pain in that country. But more recently, dezocine is being used in China for other moderate to severe pain conditions such as cancer and has been examined in clinical trials for a wide range of potential applications [10]. In this review, we focus on dezocine’s potential for causing dependence/addiction, review the medicinal chemistry of dezocine, discuss possible uses for dezocine outside of acute pain, and summarize recent efforts to formulate and modify dezocine for chronic administration.

## 2. Medicinal Chemistry of Dezocine

**Scheme 1:**
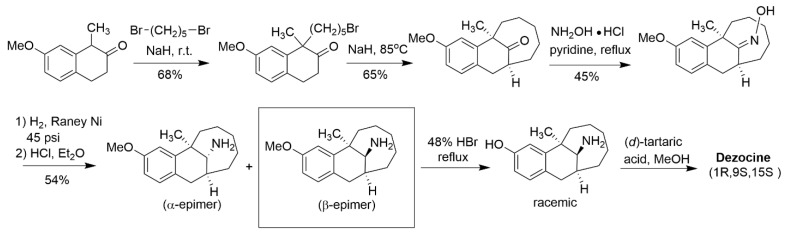
Original synthesis of dezocine described in [7] (Freed et al., 1973).

**Figure 3:**
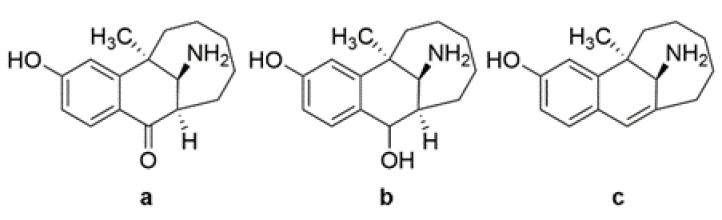
Impurities from the large-scale synthesis of dezocine ((**a**,**b**) less potent; (**c**) more potent).

Interestingly, relatively few chemistry-oriented reports around dezocine have appeared since its original disclosure. The original reported synthesis of dezocine involved seven steps (Scheme 1) and suffered from lower yields due to the need for a kinetic resolution in the last step of the synthetic scheme. In the course of large-scale syntheses of dezocine, three byproducts were produced, which were synthesized to confirm their structures (Figure 3). Two of those byproducts (Figure 3a,b) showed significantly less analgesic activity in a rat tail flick model, but the third byproduct (Figure 3c) was equipotent to morphine in that study. Since that time, a few patent applications have been filed claiming improved syntheses of dezocine, and at least four patent applications describing novel dezocine-like analogs (other than prodrugs) have appeared [11,12,13,14]. However, to our knowledge, none of these novel analogs of dezocine have advanced to clinical trials.

A number of patent applications specifically addressing the production of dezocine have been filed since the original synthesis was published. Table 1 summarizes those inventions, which include improved synthetic routes, improved synthesis of important intermediates, improved methods for isolating chirally pure products, and specific crystal forms. Despite the discontinuation of dezocine in the US in 2011, the continued patenting activity in China and expanded worldwide coverage for some applications (even as recently as 2022) indicate continued interest in dezocine.

## 3. Biased Ligand Signaling

The pharmacology of dezocine has been extensively reviewed by us and others [10,15,16,17]. It is a partial agonist for both u- and k-opioid receptors (MOR and KOR, respectively). Dezocine’s partial agonist activity at MORs at least partly explains its ceiling effect in causing opioid-associated side effects such as respiratory depression, constipation, and sedation. It also explains how dezocine is able to antagonize both the analgesic activity and adverse effects of MOR full agonists such as morphine [18]. A similar profile is seen with other MOR partial agonists such as pentazocine. However, recent data suggest that partial agonism is not the only reason for dezocine’s superior safety profile [19,20].

**Figure 4:**
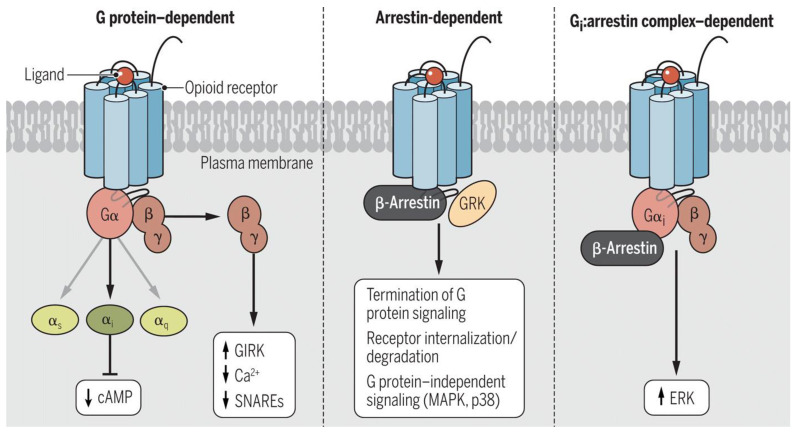
Signaling pathways used by opioid receptors. Opioid receptors signal through three distinctive pathways. (**Left**) G-protein dependent pathway, wherein the receptor engages G-proteins to initiate intracellular signaling; (**Middle**) arrestin-dependent pathway, wherein the receptor becomes phosphorylated and engages beta-arrestins, resulting in receptor desensitization and internalization; (**Right**) Gi/arrestin complex pathway, wherein the receptor forms a complex with Gi and beta arrestins, resulting in ERK-associated downstream signaling. Reproduced with permission from Che et al., 2021 [21].

Like many G-protein-coupled receptors (GPCRs), opioid receptors can signal through a second pathway involving beta-arrestins (Figure 4) [21,22]. In this pathway, the binding of a ligand to the receptor recruits kinases that phosphorylate the receptor, resulting in the recruitment of beta-arrestins that desensitize the receptor and target it for internalization. However, other kinase-associated pathways are activated by beta-arrestin, making the process more complex than just receptor regulation and blockade. MORs signal through both the GPCR and beta-arrestin pathways (Figure 3). The arrestin pathway has been implicated in a number of deleterious aspects of MOR agonists. As one might expect, opioid tolerance is caused by arrestin-associated receptor desensitization and internalization [23]. However, there is evidence that the beta2-arrestin pathway also plays a role in many of the adverse effects associated with opioid analgesics [24], including respiratory depression, constipation, and even addiction and dependence [25].

**Figure 5:**
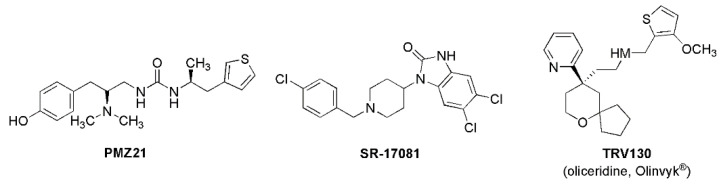
Structures of selected MOR biased agonists.

Dezocine is a biased signaling ligand at μ-opioid receptors (Figure 4). The analgesic activity of MOR agonists is mediated by G-protein signaling, while many of the undesirable side effects of MOR full agonists, such as respiratory depression, constipation, sedation, and addiction, are thought to be mediated through β-arrestin signaling [26]. Dezocine has been shown to be a biased ligand that signals primarily through G-proteins [27], which may at least partially explain its reduced propensity for typical μ-opioid side effects. In animal studies, dezocine showed a lower incidence of constipation compared to morphine, codeine, and d-propoxyphene [28]. Dezocine also produced a significantly lower incidence of respiratory depression compared to morphine in rhesus monkeys. A similar safety profile was reported for dezocine in humans [29]. Dezocine induced some respiratory depression in humans at super-clinical doses but exhibited a ceiling effect, with no further respiratory depression observed at doses exceeding 0.3 mg/kg i.v [30]. Only a limited amount of sedation is observed at clinical doses [31]. This realization has stimulated the search for MOR-biased opioids. A number of them have been identified (Figure 5), including the marketed analgesic oliceridine (Olinvyk^®^, TRV-130) [21,32,33,34]. However, the situation seems to be more complicated than simply a case of receptor bias. Despite its bias for MOR GPCR signaling and reduction in some opioid-associated side effects, oliceridine is a full agonist at the MOR and still generalizes to morphine. The package label comes with warnings about abuse, addiction, and withdrawal [35].

## 4. Dezocine and Biogenic Amine Reuptake

**Figure 6:**
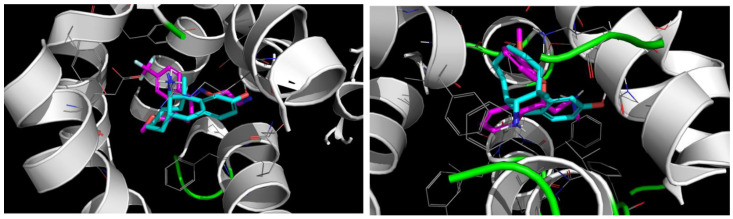
Docking models of dezocine (Cyan) with the human serotonin and norepinephrine transporters. (**Left**) Dezocine is predicted to occupy the same binding site as the SSRI fluvoxamine (magenta, docking model using the co-crystal structure of fluvoxamine bound to the human SERT, PDB 6AWP, Coleman and Gouaux, 2017) [37]; (**Right**) dezocine is predicted to occupy the same binding site as the NRI reboxetine (magenta, docking model using the cryo-EM structure of reboxetine bound to the human NET, PDB 8ZP1, Song and Wu, 2024 [38]).

In addition to its activities at opioid receptors, dezocine displays inhibitory activity against the serotonin reuptake transporter (SERT) and the norepinephrine reuptake transporter (NET) [36]. Through binding to the site occupied by known antidepressant reuptake inhibitors (Figure 6). Dezocine possesses weaker affinity for the SERT (PKi = 6.96) compared to potent selective serotonin reuptake inhibitors (SSRI) such as paroxetine and escitalopram (PKis ~ 9.0) and weaker affinity for the NET (PKi = 6.00) compared to potent norepinephrine reuptake inhibitors (NRI) like nisoxetine (PKi = 9.15). Nevertheless, for a common clinical dose of dezocine (0,15 mg/kg, i.v.) in a 70 kg human with approximately 5.7 L blood volume, the free unbound exposure of dezocine based on its 80% protein binding and assuming a brain/plasma ration of 1.0 ((the brain to plasma ratio for dezocine has never been reported; this speculated number is compared to a B/P ratio of 0.6 for morphine and 1.9 for heroin) is around 1.6 micromolar, which covers the Ki for NET. Thus, it is anticipated that the effects of at least NET inhibition should be realized with clinically relevant doses of dezocine.

The NET inhibitory activity has implications for the analgesic efficacy of dezocine. Spinal norepinephrine is known to be involved in both acute and chronic/neuropathic pain perception via α2A receptors [39]. Inhibitors of NET have been shown to be effective against a number of pain conditions (summarized in Childers and Abou-Gharbia, 2021 [15]). A number of studies demonstrated that at least part of dezocine’s analgesic effects in animal models of neuropathic pain were associated with the inhibition of norepinephrine reuptake in the spinal cord [40]. Thus, this additional analgesic mechanism is another factor that distinguishes dezocine from many other opioid modulators [41]. Evidence from both animal models [42,43,44] and human conditions [19,45,46,47] support this supposition. Recently, data suggested that dezocine may exert some of its antihypersensitivity effects through the suppression of the Akt1/GSK-3β pathway, although this hypothesis needs further investigation [48].

Dezocine is reported to show antidepressant-like activity in animal models [49,50]. Reports of human antidepressant activity have also appeared [51]. KORs are involved in depression, especially withdrawal-associated depression following the discontinuation of MOR agonists [52,53]; however, α2A-noradrenergic agonists also display efficacy in reducing opioid withdrawal symptoms [54]. Since the inhibition of norepinephrine uptake enhances adrenergic tone, reuptake inhibition would be expected to show activity similar to that of α2-agonists. Thus, dezocine’s combination of KOR partial agonism and norepinephrine reuptake inhibition suggests a possible role for the drug in reducing the withdrawal symptoms associated with opioid discontinuation. Indeed, this use has recently been proposed, as discussed below.

## 5. Dezocine and Addiction

Opioid dependence and addiction are both complicated in their neurobiology [55]. Dezocine is not classified as a controlled substance by the World Health Organization. There are no reports of dezocine abuse or addiction in the English language literature and only a few reports of concern for dezocine abuse in the Chinese language literature [56]. That is not to say that dezocine is devoid of the euphoric effects associated with abused MOR agonists such as morphine, fentanyl, and oxycodone. Human substance abusers described dezocine as feeling “similar to dope” (a slang term for substances of abuse, specifically morphine) in a 1985 study [57]. However, the few reports of withdrawal from chronic dezocine use described the symptoms as short term and mild compared to those of traditional opioids [58,59].

Results in animal studies corroborate human experiences. Dezocine has been shown to generalize to MOR agonists in various animal models [60], but dezocine’s profile is different from that of MOR full agonists. Dependence liability studies in rhesus monkeys showed that dezocine neither acutely substituted for morphine in self-administration studies nor produced dependence after chronic administration [28]. In codeine-addicted macaque monkeys, dezocine substituted for codeine in self-administration studies but with a much shallower dose-response curve and an apparent ceiling effect compared to other opioids [61]. In drug discrimination studies performed in macaque and squirrel monkeys, dezocine generalized to morphine and fentanyl in some cases [61,62] but not in others [28]. The MOR antagonist nalorphine blocked the discriminative effects of all three drugs from saline in multiple studies. Morphine and fentanyl fully substituted for dezocine in pigeons [63]. Discrimination of all three compounds from saline was reversed by treatment with the MOR antagonist nalorphine, although much higher doses of the antagonist were required to block the effects of dezocine in pigeons compared to the two full MOR agonists. A KOR antagonist had no effect on the discriminative activity of dezocine. These data strongly suggest that dezocine displays a lower risk for addiction compared to traditional MOR agonists.

These data raise the question of why dezocine seems to be less addictive than other MOR agonists like fentanyl, morphine, and oxycodone. Dezocine is a partial agonist at MOR2 rather than a full agonist like addictive opioids. However, the MOR partial agonists that are used to treat opioid use disorder, such as buprenorphine, still cause addiction. Many patients using such agents to withdraw from, for example, fentanyl abuse, spend much of their lives dependent on those partial agonists. Dezocine is a weak partial agonist/antagonist at KORs. But drug discovery research on KOR antagonists has failed to demonstrate efficacy for such agents as anti-addiction drugs [64].

Part of the answer may lie in the fact that dezocine is a biased agonist that signals only through G-proteins. Research into the role of opioid signaling in addiction supports the hypothesis that beta2-arrestin plays a significant role in the neurochemical changes that lead to addiction. Beta2-arrestin engagement leads to the internalization of MORs and opioid tolerance, but tolerance is not the same as dependence. However, both animal and human studies suggest that β2-arrestin engagement plays a role in the behavioral effects of multiple classes of abused drugs, including opioids, cannabinoids, stimulants, and possibly even nicotine and alcohol [65]. These pro-addictive effects are mediated through dopamine receptors, which are well known to play a role in reward and dependence [66]. However, as discussed above, the recently approved G-protein-biased MOR agonist oliceridine still possesses a risk of dependence and withdrawal. Oliceridine is a full agonist MOR. So, it may be dezocine’s unique combination of pharmacological properties that bestow it with a lower risk of becoming addicted.

## 6. Dezocine and Lack of Withdrawal Symptoms

Recently, there has been interest in exploring the use of dezocine to help people with an addiction taper off opioids [56,67]. But evidence suggests that dezocine may not just act as a less efficacious opioid; it may prevent the withdrawal symptoms. Like the opioid antagonist nalophine, dezocine reversed morphine-induced narcosis and loss of righting reflex in rats. In morphine-dependent rats, dezocine alleviated withdrawal symptoms, inhibited the reinstatement of conditional place preference, and prevented morphine-stimulated astrocyte activation and KOR internalization [68]. Depression is well described as a symptom associated with opioid withdrawal [69]. As an NRI and a weak KOR partial agonist, dezocine would be expected to demonstrate antidepressant activity, and indeed, a few reports support that assertion [52,70,71]. However, agonists of α2A adrenergic receptors, such as lofexidine (Lucemyra^®^), have shown effectiveness in treating both opioid withdrawal symptoms and relapse [72]. As an NRI, dezocine would be expected to increase adrenergic tone. Thus, dezocine’s complex pharmacology might make a good candidate to help people with an addiction withdraw from opioid use.

## 7. Dezocine and Addressing Physicochemical Properties

At present, dezocine is administered in the clinical setting as an intravenous (i.v.) or intramuscular (i.m.) injection. This is due to its physicochemical and ADME properties. The molecule displays an in vivo half-life of around 2–2.5 h in humans due to rapid metabolism, which involves both CYP-450 mediated oxidative metabolism and glucuronidation of the phenolic hydroxyl group. If dezocine is ever to be used for indications that require sub-chronic administration, then other modes of administration will have to be developed, and the rapid metabolism will have to be overcome.

To date, the development of oral formulations for dezocine has met with limited success, primarily due to the high first-pass metabolism that rapidly reduces plasma levels of the drug following oral administration. Recently, patent applications have been published describing alternatives to i.v. administration, including transdermal patches and intramuscular/subcutaneous depots (Table 2).

A plethora of such inventions have appeared that generically claim dezocine as one of a number of drugs suitable for the technology described, but several inventions specifically target dezocine or provide data with dezocine. Most of these patent applications come from Chinese organizations. One recent paper from the University of Pennsylvania [73] describes a neutral formulation of dezocine employing cyclodextrin, a biodegradable cyclic oligosaccharide that serves as both a neutral solubilizing agent and a controlled release agent [74]. Dezocine in this formulation was able to reduce self-administration of oxycodone in rodents when administered intraperitoneally at doses previously shown to be effective in traditional formulations (0.5–10 mg/kg) in a volume of 30 μg/μL.

**Figure 7:**
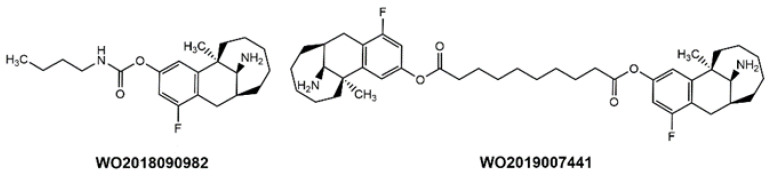
Examples of dezocine prodrugs in the recent patent literature.

A number of patent applications have also appeared addressing dezocine’s limited in vivo half-life (Table 2). These efforts include sustained-release formulations and prodrug analogs designed to interact with hydrolytic enzymes to slowly release dezocine in a controlled manner. Examples of such prodrugs, which involve ester analogs of the phenolic hydroxyl group, are provided in Figure 7.

One intriguing approach is the development of an intranasal formulation of dezocine. Until recently, dezocine’s limited aqueous solubility and requirement for acidic formulations hindered the ability to produce formulations with suitable pH and high enough drug concentration to be suitable for intranasal administration. However, in 2022, the University of Pennsylvania group published a paper describing a neutral nano-formulation of dezocine delivered intranasally that provided higher plasma and brain concentrations of the drug than an identical dose delivered intraperitoneally [27]. The cyclodextrin-based formulation described above was also shown to reduce oxycodone self-administration in rodents [73]. Thus, intranasal administration could open the way for using dezocine chronically in an out-patient setting.

Several patent applications have also appeared targeting dezocine’s limited in vivo half-life (Table 2). These efforts include sustained-release formulations and product analogs designed to interact with hydrolytic enzymes to slow the release of dezocine in a controlled manner. Examples of such prodrugs, which involve ester, amide, and carbamate analogs of the phenolic hydroxyl group, are provided in Figure 7.

## 8. Opinion: Uses of Dezocine

The use of dezocine as a non-addictive analgesic has been recently reviewed by us and others [10,15]. Dezocine is widely used as a short-acting alternative to MOR full agonists for the treatment of pain in China. As discussed above, the G-protein biased MOR partial agonist activity may be part of the reason for dezocine’s lower propensity to induce dependence, but other MOR partial agonists and other G-protein biased MOR agonists currently used to treat opioid use disorder still possess a risk of addiction. Dezocine’s NRI activity likely contributes to its anti-nociceptive activity but may play a role in the apparent lack of dependence due to the indirect increase in adrenergic tone at α2A receptors associated with inhibiting norepinephrine reuptake. Dezocine is currently off the market in the US. But recently, an international panel of policy experts, society leaders, anesthesiologists, pain experts, scientists, pharmaceutical industry leaders, entrepreneurs, and educators met to discuss issues related to perioperative pain management. One subject of the meeting was the reintroduction of dezocine to the US market [75].

As discussed above, dezocine has demonstrated efficacy in the management of neuropathic pain in both animals and the clinical setting. Data suggest that it does so by suppressing spinal hypersensitivity through both MOR and NRI pathways [40]. In fact, there is evidence that the NRI activity inherent in dezocine may actually prevent opioid tolerance that occurs with chronic use of MOR full agonist analgesics [76]. The ceiling effect seen for side effects that occur with dezocine should make it a safer alternative to full agonists like morphine. The opioid-like euphoria that some patients report after taking dezocine, albeit less intense than that of MOR full agonists, is still a concern for chronic use such as treating neuropathic. However, gabapentin, a widely used drug for treating neuropathic pain, is also reported to produce psychological effects and, unlike dezocine, can also be addictive if misused [77]. In its present formulations (i.v., i.m.), dezocine’s use as a practical anti-neuropathic agent is limited. However, the scientific and patent literature indicate that research into novel, easier-to-use administration routes is occurring. Clearly, formulations that patients can administer themselves in an out-patient setting are needed, and additional clinical trials are warranted to study the overall efficacy and safety of dezocine for treating neuropathic pain conditions. Still, the possibilities are intriguing.

As discussed above (Section 6), there is recent interest in exploring dezocine as an agent for treating opioid use disorder. Based on data from both animal and human studies, its activity as a G-protein-biased partial MOR agonist may facilitate weaning off of MOR full agonists without withdrawal-associated side effects and without inducing dependence or addiction in its own right. The NRI activity displayed by dezocine may further eliminate withdrawal side effects by increasing adrenergic tone at α2A receptors. Of course, clinical trials will be needed to validate that hypothesis, but the ability to perform those trials may be closer to becoming a reality due to the recent development of formulations that support convenient administration in an out-patient setting, such as intranasal.

## 9. Conclusions

The preclinical and clinical data accumulated to date indicate that dezocine is an effective analgesic with efficacy similar to or better than that of morphine. Its unique pharmacology makes it a safer agent for treating acute pain compared to traditional opioids. Dezocine also displays a significantly lower risk of developing dependence and addiction compared to other opioids, thanks to its G-protein-biased partial agonist activity at MORs and possibly due to its NRI activity.

There is currently a discussion among international experts on the possibility of reintroducing dezocine to the US market [75]. However, that effort would meet some competition (see Table 1 in Kingwell, 2025 [78]). At the time of this writing, the US Food and Drug Administration had just recently approved Vertex’s suzetrigine (VX-549, Journavx^®^), a selective, peripherally restricted, allosteric inhibitor of the voltage-gated sodium channel Nav1.8 [78,79]. Suzetrigine was devoid of the typical side effects associated with opioids. In phase 3 clinical trials, suzetrigine performed comparably to an opioid/acetaminophen combination in relieving pain from abdominoplasty and bunionectomy but did not differentiate itself from the placebo in a Phase 2 clinical trial for lumbosacral radiculopathy (a form of nerve damage-induced neuropathic pain). Thus, the extent of its efficacy in different pain conditions remains to be seen. Another consideration will be the willingness of insurers to cover Journavx^®^ at an estimated cost of $31/day compared to oxycodone, which costs around $2/day. It remains to be seen how broadly effective Journavx^®^ is in the general patient population and whether or not physicians worldwide are willing to move away from their traditional use of opioids for pain. But for now, the approval of Journavx^®^, the first US approval of a new analgesic in 27 years, has generated excitement as the world still suffers from the opioid crisis. Dezocine will need to differentiate itself from suzetrigine. There is some evidence that dezocine may be effective against at least some forms of neuropathic pain. Further clinical trials are needed to confirm these observations and better understand the extent of dezocine’s usefulness in this complicated therapeutic area.

The use of dezocine as an agent to support the withdrawal of addicted patients from full agonist opioids is intriguing. Unlike some of the agents used for this indication, dezocine’s lack of dependence and addiction would be a significant improvement. In addition, there is evidence that dezocine reduces or prevents opioid withdrawal symptoms. Dezocine is not devoid of the risk of respiratory depression, but other partial MOR agonists like buprenorphine also cause respiratory depression. Like those agents that are used today, dezocine displays a “ceiling effect” that limits the degree of respiratory depression that it can cause. This does not seem to be a hurdle to its use for opioid use disorder. Until recently, the need to administer dezocine i.v. or i.m. has prevented its consideration as a drug for sub-chronic use. However, recent advances such as intranasal formulations may resolve that weakness.

Dezocine remains something of a “double-edged sword”. While it does not cause dependence and addiction, it still causes euphoric effects, which could lead to misuse. This may prevent its use in conditions that require chronic administration, such as depression. However, that antidepressant activity may be part of why dezocine is effective in preventing opioid withdrawal and relapse. Thus, the data to date indicate that dezocine is an effective, non-addictive analgesic with a superior safety profile compared to other opioids. Its usefulness in other indications like neuropathic pain and opioid use disorder will need to be assessed in clinical trials before we truly know the full extent of dezocine’s benefits. But for now, it is safe to say that dezocine seems to be more friend than foe.

## Figures and Tables

**Table 1:** Patent applications describing synthetic or manufacturing improvements for dezocine. Results of a Scifinder-n^®^ search performed on 5 January 2025.

Patent/Application Number	Original Publication Date	Title	Assignee
CN101671269	17 March 2010	Preparation method of substituted methylene benzocyclodecene ketoxime	Yangtze River Pharmaceutical Group Co., Ltd., Shanghai, China
CN102503840	20 June 2012	Preparation method of dezocine	Yangtze River Pharmaceutical Group Co., Ltd., Shanghai, China
CN104910002	16 September 2015	Dezocine key intermediate preparation method	Wenzhou Medical University
CN107337609/ WO2018214877	10 November 2017	A dezocine crystal form and a preparing method thereof	Yangtze River Pharmaceutical Group Co., Ltd., Shanghai, China
CN107522625	29 December 2017	Crystal form of dezocine and preparation method therefor	Yangtze River Pharmaceutical Group Co., Ltd., Shanghai, China
CN107778187	9 March 2018	Dezocine crystal form A and preparation method thereof	Jiangsu Nhwa Pharmaceutical Co., Ltd., Nanjing, China
CN108299173/ WO2018214877	20 July 2018	Asymmetric synthesis method of dezocine key intermediate	Yangtze River Pharmaceutical Group Co., Ltd., Shanghai, China
CN110283085	27 September 2019	Dezocine production process	DR Double Crane Limin Pharamceutical Jinan Co., Ltd., Jinan, China
CN113896621	7 January 2022	Asymmetric synthesis method of dezocine key intermediate	Ningbo Zejun Pharmaceutical Tech Co., Ltd., Ningbo, China
CN113979875/ WO2022022388	28 January 2022	Dexocine derivative crystal form A as well as preparation method and application thereof	Yangtze River Pharmaceutical Group Co., Ltd., Shanghai, China
CN114380702	22 April 2022	Separation and purification method of dezocine	Taizhou Danding Biotechnology Co., Ltd., Taizhou, China
CN115043742	13 September 2022	Preparation method of dezocine	Nanjing Damei Biological Pharmaceutical Co., Ltd., Nanijng, China

**Table 2:** Recent patent applications describing improvements to dezocine’s route of administration and pharmacokinetic profile. Results of a Scifinder-n^®^ search performed on 5 January 2025. Only patent applications specifically claiming dezocine or presenting data for dezocine are included.

Patent/Application Number	Original Publication Date	Title	Assignee
**Inventions addressing improved non-intravenous administration**			
WO2009032246	12 March 2009	Compositions and methods for the delivery of poorly soluble drugs	Nanotherapeutics Inc., Alachua, FL, USA
CN104622791	20 May 2015	Dezocine oral preparation	Yangtze River Pharmaceutical Group Co., Ltd., Shanghai, China
WO2016157509	15 October 2015	Compositions and methos for treating opioid receptor associated diseases	University of Pennsylvania, Philadelphia, PA, USA
CN108210933/ WO2018113581	28 June 2018	Conjugate of dezocine and polyethylene glycol	Jenkem Technology Co., Ltd., Shanghai, China
CN113679659	23 November 2021	Dezocine in-situ gel composition and application thereof	Yangtze River Pharmaceutical Group Co., Ltd., Shanghai, China
WO2020008370A1	9 January 2020	Transdermal patch	Clexio Biosciences Ltd., Yokneam, Israel
WO2020008366A1	9 January 2020	Transdermal dosage form	Clexio Biosciences Ltd., Yokneam, Israel
CN108653446	16 October 2018	Surface anesthesia medicine composition, microemulsion, as well as preparation method and application of microemulsion	West China Hospital, Sichuan University, Sichuan, China
CN108285423	17 July 2018	Pain-easing active compound and medical application thereof	Taizhou Huayuan Medicine Technology Co., Ltd., Taizhou, China
**Inventions addressing short duration of action (e.g., sustained release formulations, prodrugs)**			
CN11689549			
CN115770232	10 Marsh 2023	Dezocine sustained release microsphere and preparation method thereof	Soochow University, Jiangsu, China
CN113730340	3 December 2021	Fatty acid sustained-release composition for injection and preparation method and application thereof	China Pharmaceutical University, Jiangsu, China
CN110101865	9 August 2019	Dezocine-containing medicine sustained-release preparation and preparation method thereof	Tianjin Third Central Hospital
CN110101660	9 August 2019	Compound medicine sustained-release preparation containing dezocine and lidocaine and preparation method thereof	Tianjin Third Central Hospital, China
WO2018090982	24 May 2018	Preparation of benzodicycloalkane derivative as analgesics	Shanghai Haiyan Pharmaceutical Technology Co., Ltd., Shanghai, China
WO2019007443	10 January 2019	Dezocine analogue ester-containing sustained-release suspension and preparation method thereof	Shandong Danhong Pharmaceutical Co., Ltd., Heze, China
WO2019007441	10 January 2019	Sustained-release suspension containing dezocine analog ester and preparation method thereof	Shandong Danhong Pharmaceutical Co., Ltd., Heze, China
CN108704137	26 October 2018	Opioid receptor partial agonist supported sustained release microsphere as well as preparation method and application thereof	Institute of Process Engineering, Chinese Academy of Sciences, China
WO2019007285A	10 January 2019	Crystal form and amorphous form of dezocine analogue hydrochloride	Shandong Danhong Pharmaceutical Co., Ltd., Heze, China
